# Zinc-based metal organic framework with antibacterial and anti-inflammatory properties for promoting wound healing

**DOI:** 10.1093/rb/rbac019

**Published:** 2022-04-18

**Authors:** Yuting Chen, Jinhong Cai, Dachang Liu, Shuhan Liu, Doudou Lei, Li Zheng, Qingjun Wei, Ming Gao

**Affiliations:** 1 Guangxi Engineering Center in Biomedical Materials for Tissue and Organ Regeneration, The First Affiliated Hospital of Guangxi Medical University, No. 22 Shuangyong Road, Qingxiu District, Nanning 530021, China; 2 Department of Orthopaedics Trauma and Hand Surgery, The First Affiliated Hospital of Guangxi Medical University, No. 6 Shuangyong Road, Qingxiu District, Nanning 530021, China; 3 Guangxi Collaborative Innovation Center of Regenerative Medicine and Medical Bioresource Development and Application, The First Affiliated Hospital of Guangxi Medical University, No. 22 Shuangyong Road, Qingxiu District, Nanning 530021, China; 4 Guangxi Key Laboratory of Regenerative Medicine, The First Affiliated Hospital of Guangxi Medical University, No. 22 Shuangyong Road, Qingxiu District, Nanning 530021, China

**Keywords:** zinc ions, metal organic framework, antibacterial, anti-inflammatory, skin wound healing

## Abstract

The synergistic effect of antibacterial and anti-inflammatory is needed to overcome the problem of wound healing difficulties. Based on the favorable antibacterial and anti-inflammatory effect of zinc ions (Zn^2+^) and the physicochemical properties of metal organic frameworks (MOFs), we prepared nanosized zinc-based MOF: Zn-BTC with the ability to slowly release Zn^2+^. In cellular levels, Zn-BTC possessed lower toxicity to fibroblasts and enhanced capacity of cell proliferation and migration. It also had good bactericidal effect on multiple drug-resistant bacteria by reducing 41.4% MRSA and 47.2% *Escherichia coli*. In addition, Zn-BTC also displayed the ability of lowering the expression of antioxidant genes: superoxide dismutase 1, superoxide dismutase 2 and interleukin 6, and enhancing the expression of wound healing genes: transforming growth factors-β and type I collagen. Finally, it also demonstrated that Zn-BTC could effectively improve the skin wound healing of SD rats and had no toxicity on major organs. The favorable biocompatibility, antibacterial and anti-inflammatory properties of Zn-BTC gave a new insight of designing novel MOFs for promoting skin wound healing.

## Graphical Abstract

**Graphical Abstract:**
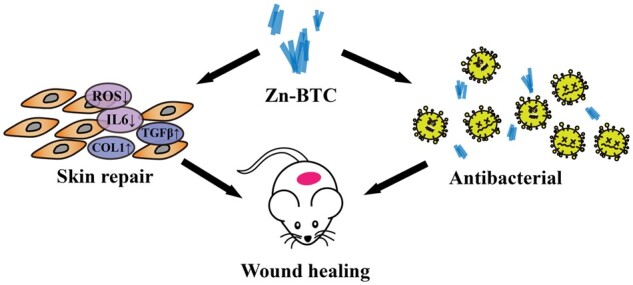

## Introduction

As the largest organ of human body, skin is the first defense barrier of the human body [1]. Once damaged by physiological diseases, the barrier will be broken, seriously affecting people’s physical and mental health. The exposed derma of skin wound surface can be easily damaged by many pathogenic factors like infection and inflammation [2], which finally delays the wound healing and further leads to septicemia [3, 4]. Therefore, anti-microbial is of significance in skin wound healing.

Although the use of antibiotics can effectively reduce the probability of bacterial infection, the effectiveness is declining yearly with the emergence of multiple drug-resistant bacteria [5], which brings challenges to the development of medical antibacterial materials. Meanwhile, during the healing process of infected wounds, normal redox signals are destroyed, leading to increased reactive oxidative stress (ROS) levels in wound areas [6], and the subsequent impaired antioxidant defense function [7]. And oxidative stress occurs when ROS over produced, resulting in impaired function of skin fibroblasts and keratinocytes [7]. Many studies have pointed out that ROS is closely related to inflammation [8, 9], which have various adverse effects on wound healing [10–12]. Therefore, an effective strategy must be explored to simultaneously improve the antibacterial, ROS scavenging and anti-inflammatory capacity to promote skin wound healing.

Metal organic frameworks (MOFs) are known as a class of porous materials consisted of organic ligands connected with inorganic metal ions or clusters [13–16]. Current research have focused on developing antibacterial MOFs by adjusting the connection points of metals, where the gold, silver, zinc, iron, magnesium, manganese and copper are widely applied to prepare the novel MOF for antibacterial therapy [17]. Among them, zinc, as a trace element required by the body, has good biocompatibility and is easily metabolized without causing cumulative damage [18, 19]. Therefore, zinc-based MOF can be utilized as an ideal antibacterial material.

In addition, zinc ion (Zn^2+^) also has many irreplaceable biological advantages. As a well-known antibacterial agent, Zn^2+^ has good antibacterial activity against viruses, fungi, gram-bacillus and other bacteria [20, 21]. By inhibiting manganese uptake and disrupting carbon metabolism, overdose extracellular Zn^2+^ leads to a notable toxic effect on bacterial [21]. In fact, excessive transmission of Zn^2+^ to kill bacteria is one of the body’s defense mechanisms against bacteria [22–24]. Furthermore, Zn^2+^ also participates in the regulation of Cu/Zn superoxide dismutase (SOD) activity [25, 26], and indirectly regulates the scavenging of intracellular ROS. Studies have pointed out that Zn^2+^ consumption and loss of Cu/Zn SOD activity were related to inflammation [23–26]. Therefore, the introduction of exogenous Zn^2+^ can achieve anti-inflammatory function by restoring SOD activity. In the meantime, Zn^2+^ can also inhibit NF-κB activation, and regulation of inflammatory cytokines [27–30]. Significantly, Zn^2+^ also takes part in many wound healing processes [30, 31], including inducing vascular endothelial growth factor and angiogenesis [32], and regulating the expression of extracellular proteins, such as collagen and keratin [33]. Altogether, Zn^2+^ and zinc oxides have been confirmed the capacity of promoting wound healing [34]. However, large amounts of Zn^2+^ exposure in a short space of time can be toxic [35], and the rapid metabolism of Zn^2+^ also equals to the rapid disappearance of local antibacterial capacity. The relative stable frame structure of zinc base MOF makes it possible to degrade slowly in aqueous environment. With the slow degradation of MOF materials, the long-term release of metal ions can be achieved [15, 36], which greatly reduces the toxicity of metal ions and prolongs the function time.

**Figure 1.**
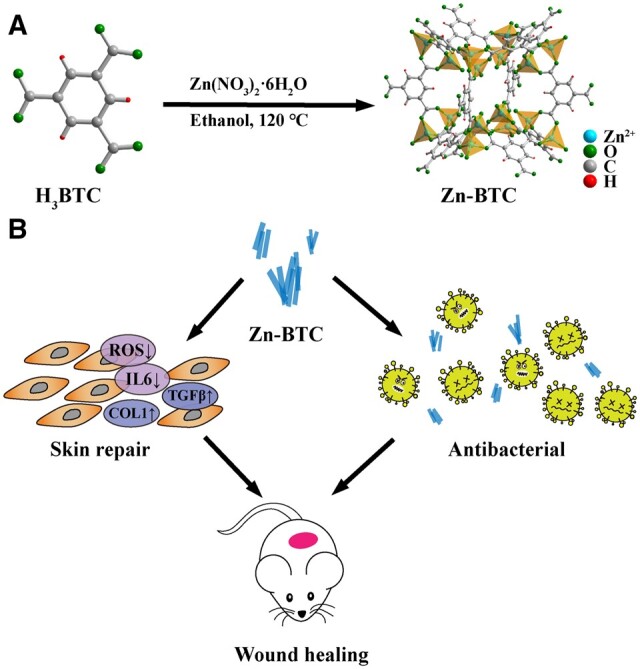
Schematic illustration of the fabrication of Zn-BTC (**A**), and its possible antibacterial and anti-inflammatory capacity, further leading to promote skin wound healing (**B**)

In this work, Zn based MOF (Zn-BTC) was prepared as white powders for improving skin wound healing (Fig. 1). Zn-BTC was synthesized by mixing the Zn(NO_3_)_2_ and trimesic acid (H_3_BTC) with thermal heating, and further characterized to confirm its successful fabrication and relative properties. The biocompatibility, antibacterial, antioxidant and anti-inflammatory ability of Zn-BTC were evaluated *in vitro* and *in vivo*. This work could provide a reference and guidance of designing novel MOF for promoting skin wound healing.

## Materials and methods

### Materials

Zinc nitrate hexahydrate [Zn(NO_3_)_2_·6H_2_O], H_3_BTC and ethanol were purchased from Aladdin Biochemical Technology Co., Ltd (Shanghai, China). Cell counting kit-8 (CCK-8) and live/dead cell kit was commercially purchased from Beyotime Biotechnology (Shanghai, China). Tryptone soy agar, hematoxylin–eosin staining kit, Masson’s trichrome (Masson) staining kit, trypsin-ethylene diamine tetra acetic acid (EDTA) solution and phosphate buffer saline (PBS) were purchased from Solarbio Science and Technology Co., Ltd (Beijing China). All chemicals were used directly without further purification and all kits were applied by following the protocols of manufactures.

### Preparation of Zn-BTC

Zn-BTC was prepared by hydrothermal method. In Brief, 1.5 g Zn(NO_3_)_2_·6H_2_O was dissolved in ethanol (25 ml) evenly with sonication, and 0.63 g H_3_BTC was dissolved in ethanol (35 ml), respectively. Then, the two solutions were mixed evenly and transferred into a 100 ml stainless steel pressure autoclave, and the mixture reacted at 120°C for 12 h. And the white precipitate was collected by centrifuge and washed for three times by ethanol. After vacuum drying, the final product was isolated as white powders.

### Basic characterization of Zn-BTC

The samples were characterized by Fourier transform infrared spectroscopy (FTIR, SHIMADZU, Japan), X-ray diffraction (XRD, Rigaku, Japan) and X-ray photoelectron spectroscopy (XPS, Thermo, UK), respectively. And the zeta potential of Zn-BTC was determined by zeta sizer (Malvern, UK). The morphology of Zn-BTC was investigated by transmission electron microscopy (TEM, H-7650, Hitachi, Japan). Besides, the thermal stability of samples was implemented by thermogravimetric analysis (TGA, TGA Q500, Swiss). To determine the pore diameter and element contents of Zn-BTC, the Brunauer Emmett and Teller (BET, Micromeritics ASAP, USA) and inductively coupled plasma-optical emission spectrometer (ICP-OES, Varian, 720-ES, USA) was applied.

### Degradation of Zn-BTC

The degradation property of Zn-BTC was indirectly evaluated by the release of Zn^2+^ in aqueous solutions. And the release of Zn^2+^ was detected by using dithizone as the indicator. The weak acidic inflammatory microenvironment (pH = 5.5) was simulated by mixing PBS buffer (pH = 7.4) with 4-morpholine ethane sulfonic acid buffer. Briefly, 2 mg Zn-BTC was immersed in 1 ml PBS buffer (pH = 5.5 and 7.4) with shaking. The samples (30 µl) at defined time points (0, 1.5, 3, 6, 12, 24 and 48 h) were collected after centrifuge and then mixed with 600 μl sodium acetate solution (containing 5% sodium thiosulfate) and DMSO solution (containing 0.05% dithizone) with the volume ratio of 1:1. After vortex mixing and waiting for 15 min, 100 μl mixture was taken and added into a 96-well plate. Finally, the absorbance was observed at 535 nm by microplate reader and the content of Zn^2+^ was determined by comparing with the standard curve. Combined with the results of ICP-OES, the release rate of Zn^2+^ could be obtained.

### Isolation and culture of dermal fibroblasts

Dermal fibroblasts were isolated from the skin tissue of Sprague-Dawley (SD) rats at the age of 3 days. Details were: the newborn SD rats were sacrificed by an over dose injection of anesthetic. And the skin tissue from back was harvested, and the fascia and adipose tissue were snipped off. Later, the tissues were then minced and treated with trypsin-EDTA solution under 37°C for 30 min. And then, the tissues were soaked in the low glucose Dulbecco’s modified eagle medium (DMEM) containing type I collagen (COL1) (1 mg/ml) and 1% penicillin/streptomycin before incubated under 37°C with 5% CO_2_ atmosphere for another 3 h. Finally, the cells were collected, washed with DMEM and plated into a 10 cm culture dish.

The fibroblasts were cultured with DMEM medium containing 10% fetal bovine serum as well as 1% penicillin/streptomycin under 37°C and 5% CO_2_ atmosphere. And the medium was replaced every 3 days. When reaching 70–80% confluence, the cells were treated with 2.5 ml trypsin-EDTA solution for digestion and subculture.

### Cell viability and cytotoxicity assay

Dermal fibroblasts cells were seeded in 96-well plate with 3000 cells per well for incubation. After cultured for 24 h, the cells were treated with samples with different concentrations for another 24 h. The cell viability was then determined by using CCK-8 kit, and the results were normalized with control group. Besides, the cytotoxicity of samples was measured through live/dead cell staining. Briefly, the fibroblasts were seeded on a 6-well plate with 3 × 10^6^ cells per well. After being co-cultured with different samples for 24 h, the fibroblasts were stained with staining solution containing 4 μM propidium iodide and 2 μM calcein-AM in PBS buffer for 5 min at 37°C. Then, the fibroblasts were rinsed with PBS buffer and imaged by fluorescent microscopy (OLYMPUS BX53F, Japan).

### Cell scratch testing

The fibroblasts were grown in 6-well plate, and a scratch wound in fibroblasts, with a confluent monolayer surface, was created using a 10-μl pipette tip. Later, fibroblasts were incubated with samples (50 μg/ml) at 37°C. And blank group was only treated with medium. After 24 h, the images of scratch wounds were taken by using a Leica DMIL LED microscope at 0 and 24 h. The result was analyzed by Image J.

### Antioxidant, anti-inflammatory and healing capacity investigation

The antioxidant and anti-inflammatory capacity of Zn-BTC was investigated in cellular levels. In brief, the fibroblasts were seeded on a 6-well plate (3 × 10^6^ cells per well) and cultured for 24 h before treated with 250 µM H_2_O_2_ for 24 h by following the previous reported work [37], and then incubated with 50 μg/ml samples for another 24 h. The normal fibroblasts were used as blank group and the fibroblasts only treated with 250 µM H_2_O_2_ were used as control group. The superoxide anion levels of fibroblasts were detected using superoxide anion fluorescence probe (Beyotime, China). In brief, after co-culture, fibroblasts were incubated with serum-free medium with dihydroethidium at 37°C for 30 min in the dark. Images were eventually observed by fluorescence microscope (Olympus, Japan), and the corresponding intensity was quantified by Image J. Further, the expression of antioxidant and anti-inflammatory related genes in each group was investigated through qRT-PCR.

Besides, the wound healing effect of Zn-BTC was investigated through qRT-PCR. Here, the normal untreated fibroblasts were used as control group for investigating skin healing genes. After co-culture with different materials, the total RNA of each group was extracted with RNA extraction kit (Magen, China) following the manufacturer’s instruction, and then applied to implement qRT-PCR. The detailed primer sequences were listed in Table 1.

**Table 1.** Detailed primer sequences for qRT-PCR

Gene	Forward sequences (5′–3′)	Reverse sequences (5′–3′)
GAPDH	TCCAGTATGACTCTACCCACG	CACGACATACTCAGCACCAG
SOD1	ATTCACTTCGAGCAGAAGGCA	ATTGCCCAGGTCTCCAACAT
SOD2	GTGTCTGTGGGAGTCCAAG	TGCTCCCACACATCAATCCC
IL6	ACAAGTCCGGAGAGGAGACT	ACAGTGCATCATCGCTGTTC
TGF-β	AGGGCTACCATGCCAACTTC	CCACGTAGTAGACGATGGGC
COL1	GATCCTGCCGATGTCGCTAT	TGGTCCATGTAGGCTACGCT

### SOD activity investigation

The fibroblasts were seeded on a 6-well plate (3 × 10^6^ cells per well) and cultured for 24 h. The cells were then treated with 100 µM H_2_O_2_ for 24 h followed by incubating with 50 μg/ml samples for another 24 h. And the normal fibroblasts and the fibroblasts treated with 250 µM H_2_O_2_ were used as blank group and control group, respectively. After co-culture, the proteins of each group were extracted with cell lysis buffer (Beyotime Biotechnology, Shanghai, China) according to the manufacturer’s instruction. Then, the intracellular SOD activity levels were evaluated by using a Cu/Zn SOD assay kit (Beyotime Biotechnology, Shanghai, China) according to the manufacturer’s instruction.

### Antibacterial capacity testing

The antibacterial ability of Zn-BTC was qualitative investigated through the co-culture of materials with bacteria. The methicillin-resistant *Staphylococcus aureus* (MRSA) and *Escherichia coli* HB101 (RP4) were incubated overnight in LB medium at 37°C. Then, the bacteria were centrifuged, rinsed and dispersed with PBS buffer. Next, the bacteria were cultured in 50 µg/ml of H_3_BTC and Zn-BTC, respectively, while the bacteria were cultured in PBS buffer as control group. The culture was implemented at 37°C with shaking for 2 h. Finally, 100 µl of bacterial solution was diluted on LB agar plates and incubated at 37°C for 24 h to measure the number of colonies on the plates.

### *In vivo* wound healing evaluation

All animal procedures were approved by Animal Ethics Committee of Guangxi Medical University and performed strictly in accordance with the guidelines. The SD rats were anesthetized by intraperitoneal injection of 10 wt% chloral hydrate and the back skin of rats was shaved and cleaned. The wound of SD rats created with a diameter of 15 mm and infected with 200 µl of MRSA suspension (1 × 10^8^ CFU/ml). After 30-min inoculation, samples were placed onto the wound sites. The healing effects were finally determined by the quantification of wound area obtained at Days 0, 3, 7 and 14 post surgery, and compared with the initial wound area at Day 0.

Besides, the infected skin wounds were harvested at Days 3, 7 and 14, and fixed in 4% paraformaldehyde (PFA) solution at 4°C overnight before being embedded in paraffin. And the paraffin blocks were cut into sections of 5 µm with a microtome before implementing H&E and Masson staining (Fast Green method). Subsequently, immunohistochemical (IHC) staining was performed by using antibody solution contained rabbit anti-interleukin 6 (IL6) (Beyotime, Shanghai, China) according to standard protocols. All images were obtained by using an optical microscopy for the determination of wound healing effects.

### *In vivo* toxicity evaluation

After treatment, the major organs of rats including heart, liver, spleen, lung and kidney, were harvested at Day 14 and fixed in 4% PFA solution followed by being embedded in paraffin for pathological slides’ preparation. After H&E staining, the sections were imaged by an optical microscope.

### Statistical analysis

All experiments were carried out at least in triplicates. Prism 6 software (GraphPad Software Inc., San Diego, CA) was applied to perform statistical analysis and create graphs. Data were presented as mean ± standard deviation. Unless otherwise stated, the statistical significances of results were determined and analyzed using one-way analysis of variance followed by the Student–Newman–Keuls posttest for normally distributed data sets, and bars represent mean ± SD).

## Results and discussion

### Preparation and characterization of Zn-BTC

**Figure 2.**
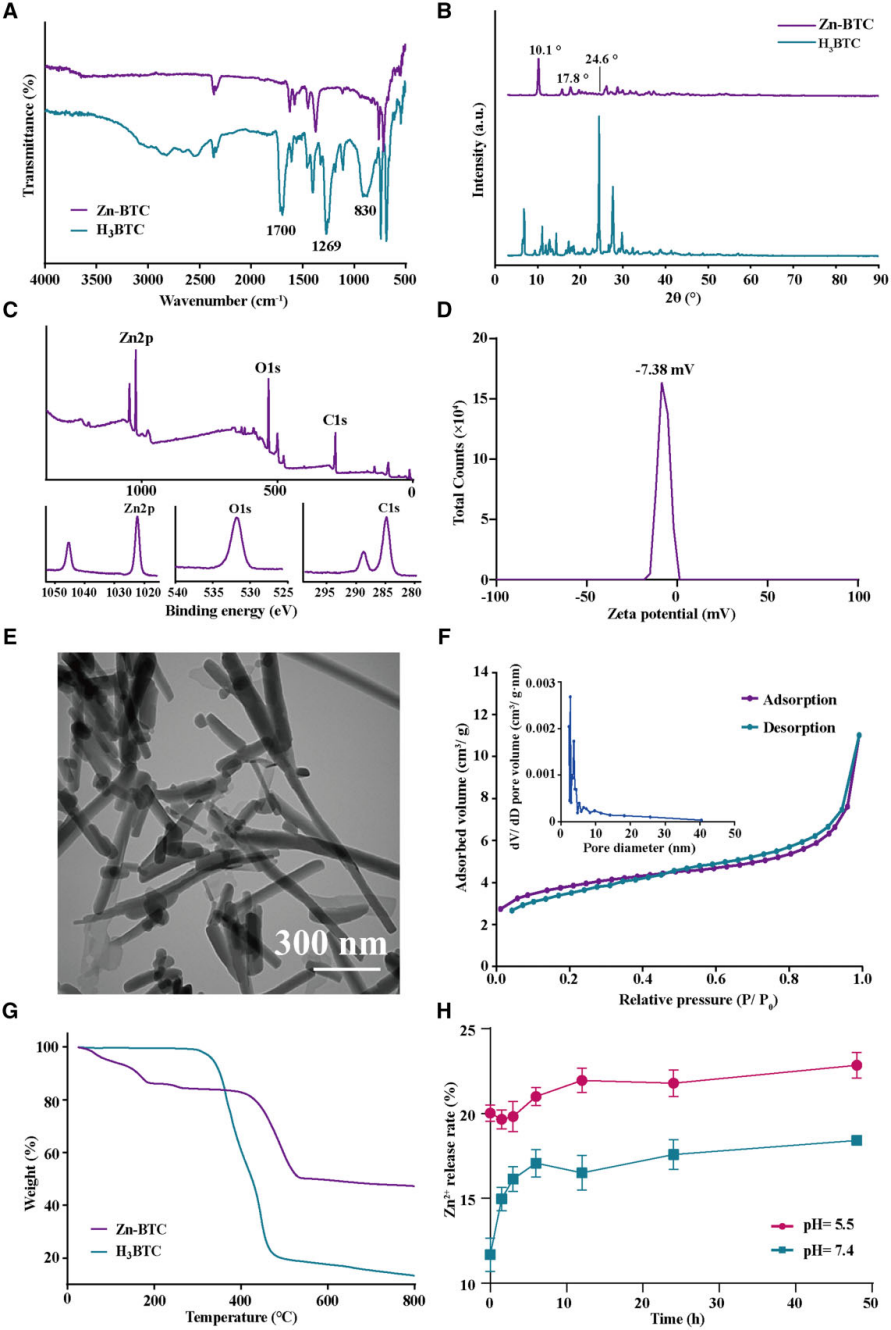
Basic characterization of Zn-BTC and H_3_BTC: FTIR (**A**), XRD (**B**), XPS: full spectrum, and Zn, C and O spectrums (**C**), zeta potential (**D**), TEM (**E**), BET (**F**), TGA (**G**) and Zn^2+^ release rate (**H**)

Zn-BTC was synthesized by mixing H_3_BTC and Zn(NO_3_)_2_·6H_2_O in ethanol solution after heating for a certain of time (Fig. 1A). After purification, the white Zn-BTC was obtained for further characterization. Figure 2A illustrated the FTIR results of H_3_BTC and Zn-BTC. As shown in Fig. 2A, the broad absorption bands observed at 3500–2400 cm^−1^ for H_3_BTC was attributed to stretching vibration of OH-while it disappeared for Zn-BTC, due to the combination between Zn^2+^ and hydroxyl groups. The observed peaks at 1700, 1269 and 830 cm^−1^ corresponding to protonated carboxyl groups of H_3_BTC were also not observed for Zn-BTC, indicating that H_3_BTC was completely deprotonated to form the bridging ligands of Zn-BTC. The XRD results of H_3_BTC and Zn-BTC were shown in Fig. 2B. The diffraction pattern for Zn-BTC displayed good crystallinity as the pronounced and sharp peaks observed. And it was also observed that Zn-BTC possessed similar crystallinity as that of H_3_BTC, and the unique peaks at 10.1°, 17.8° and 24.6° was observed for Zn-BTC, consistent with other reported work and confirmed the successful preparation of Zn-BTC [13, 33, 34]. Besides, Zn-BTC was characterized by XPS. As indicated in Fig. 2C, the Zn, C and O elements were obviously observed in Zn-BTC. Additionally, for Zn spectrum, the apparent two peaks (1022.3 and 1045.3 eV) were assigned to the 2p orbit of Zn element. Based on the XPS results, the surface elemental composition was listed as following: C (54.77%), O (33.73%) and Zn (11.5%), also indicating the successful fabrication of Zn-BTC.

The stability of Zn-BTC was characterized by Zeta sizer. As illustrated in Fig. 2D, the zeta potential was −7.38 mV for Zn-BTC, representing a certain of stability. The morphology of Zn-BTC was examined by TEM. As shown in Fig. 2E, Zn-BTC presented a rod-like shape with a length of hundreds nanometer. Besides, the pore size and its distribution of Zn-BTC were investigated by BET. Figure 2F illustrated the N_2_ adsorption–desorption isotherm of Zn-BTC. It was observed that the N_2_ uptake exponentially increased with the increase of relative pressure. After statistic calculation, the surface area and t-Plot micropore volume of Zn-BTC was 13.0129 m^2^/g and 0.0037 cm^3^/g. And the adsorption and desorption average pore was 5.2367 and 5.1889 nm, respectively. Thus, Zn-BTC presented a low surface area and favorable pore distribution, indicating the relative stable framework of Zn-BTC.

Besides, the thermal properties of Zn-BTC were investigated by TGA under N_2_ flow (20 ml/min) from 25 to 800°C. In Fig. 2G, the glass transition temperature (*T*g) was around 320°C for H_3_BTC, which moved to around 410°C after forming MOF. Meanwhile, the weight loss was close to 100% for H_3_BTC and it was only 57% for Zn-BTC. Therefore, the *T*g of Zn-BTC increased and its weight loss decreased compared to H_3_BTC, indirectly confirming the successful preparation of Zn-BTC. By ICP-OES, the zinc content of Zn-BTC was 28.72 ± 0.6%.

The degradation ability of Zn-BTC was indirectly demonstrated by the release of Zn^2+^ under different pH environment. As indicated in Fig. 2H, the rapid rise of Zn^2+^ concentration was displayed under weak acidic condition (pH = 5.5) corresponding to infection microenvironment [38], indicating that Zn-BTC degraded rapidly in this situation. The high concentration of Zn^2+^ existed in the infection and inflammation acidic microenvironment, which was conducive to achieve efficient therapy. Under pH = 7.4, the concentration of Zn^2+^ would gradually increase versus time, indicating that MOF degraded slowly and it could realize a slow and long-term release of Zn^2+^.

### Biocompatibility of Zn-BTC

**Figure 3.**
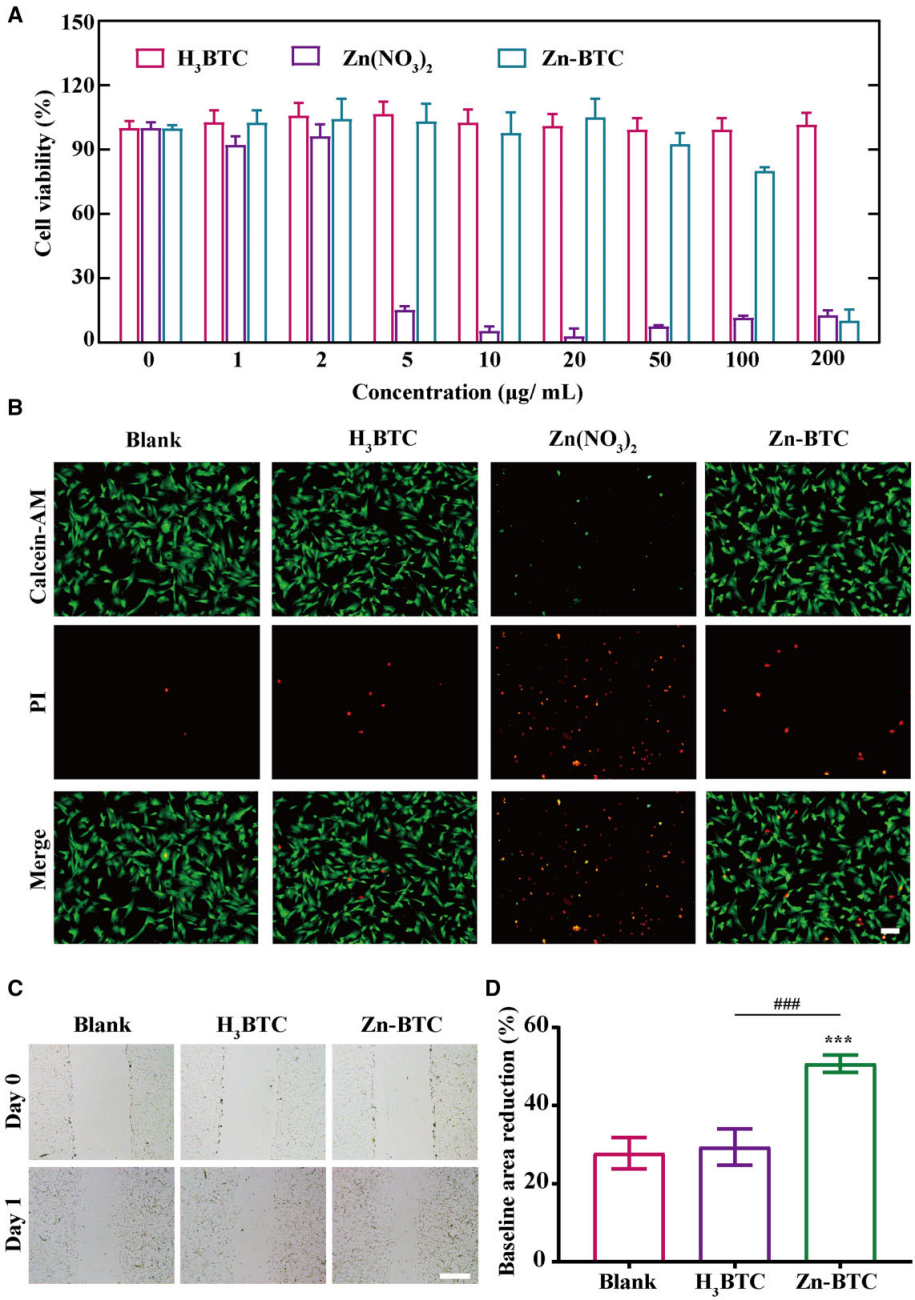
Biocompatibility of Zn-BTC. (**A**) Cell viability of H_3_BTC, Zn(NO_3_)_2_ and Zn-BTC with different concentrations by CCK-8. (**B**) Live/dead staining of fibroblasts with 20 μg/ml H_3_BTC, Zn(NO_3_)_2_ and Zn-BTC, respectively (bar =200 µm). (**C**) Cell scratch testing results of fibroblasts (bar =250 µm) and the corresponding quantified results (**D**) (‘^*^’ symbol compared with blank group, ^***^P < 0.001, and ‘^#^’ symbol compared with H_3_BTC group, ^###^*P* < 0.001)

The biocompatibility of Zn-BTC was assessed by CCK-8 assay and live/dead staining. As shown in Fig. 3A, it was shown that H_3_BTC had a low toxicity at a relatively high concentration of 200 μg/ml. However, the viability of fibroblasts cells began to decrease at the concentration of 50 μg/ml for Zn-BTC. Most significantly, the cell viability of Zn(NO_3_)_2_ obviously decreased at 5 μg/ml. Similarly, in Fig. 3B, 50 μg/ml Zn(NO_3_)_2_ presented apparent cytotoxicity for fibroblasts with a lot of dead cells (red) while only a few of fibroblasts were dead observed for Zn-BTC. The high concentration of Zn^2+^ could inhibit the viability of fibroblasts. Furthermore, Zn-BTC gradually released Zn^2+^ at a slow speed through its degradation, which significantly reduced its cytotoxicity. Therefore, it was impossible to directly use huge amount of free Zn^2+^, which caused serious damage to healthy cells. Therefore, Zn(NO_3_)_2_ would not be considered in the following experiment. It also demonstrated that high concentration of Zn^2+^ was harmful to healthy tissue and even delayed wound healing [35]. By comparing the difference between H_3_BTC and Zn-BTC, it was clarified that the sustained release of Zn^2+^ was achieved for Zn-BTC in cellular level.

Cell scratch testing was always applied to investigate the proliferation and migration of cells. As illustrated in Fig. 3C, it presented better cell migration and proliferation ability after treated with 50 μg/ml of Zn-BTC, compared to blank group and H_3_BTC. Moreover, the cell migration ratio was defined as non-migrated area divided by the baseline area. After calculation, the average migration ratio was 50.6% for Zn-BTC while it was 29.8% for H_3_BTC (Fig. 3D). The existence of Zn^2+^ could promote the migration of fibroblasts, consistent with studies previous reported [39, 40].

### *In vitro* anti-inflammatory and wound healing capacity

**Figure 4.**
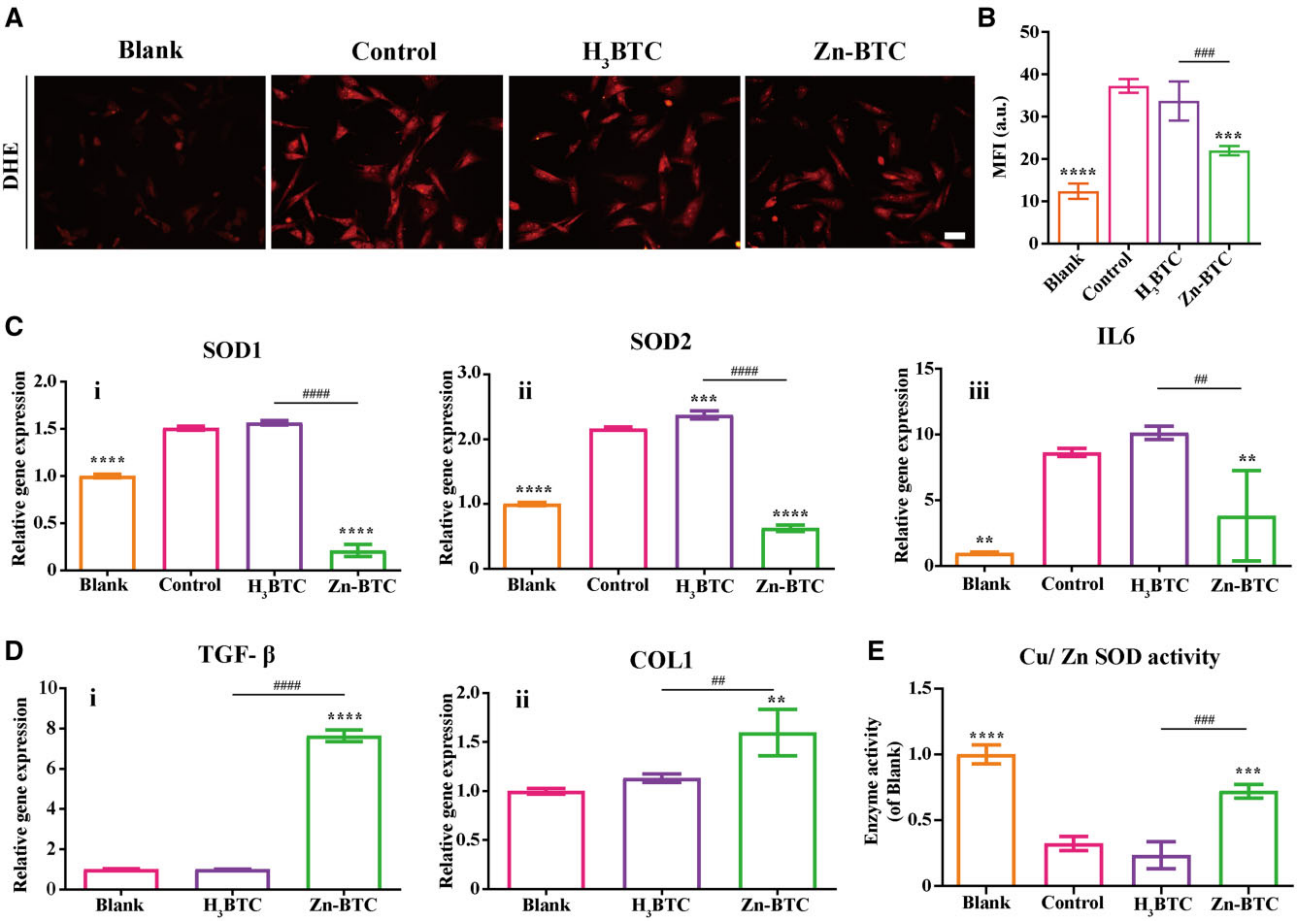
*In vitro* antioxidant, anti-inflammation, and wound healing effects on fibroblasts. (**A**) The intracellular superoxide anion levels of H_2_O_2_ stimulated fibroblasts after treated with different materials (bar =100 µm) and the corresponding quantification results (**B**). (**C**) The anti-inflammation gene expression: SOD1 (i), SOD2 (ii) and IL6 (iii) of H_2_O_2_ stimulated fibroblasts after treated with different materials. (**D**) The wound healing gene expression: TGF-β (i) and COL1 (ii) of normal fibroblasts after treated with different materials. (**E**) The quantification of Cu/Zn SOD activity (‘^*^’ symbol compared with control group, ^**^P < 0.01, ^***^P < 0.001 and ^****^P < 0.0001, and ‘^#^’ symbol compared with H_3_BTC group, ^##^*P* < 0.01, ^###^*P* < 0.001 and ^####^*P* < 0.0001)

Zinc is a structural constituent of Cu/Zn SOD and is closely related to its activity. Cu/Zn SOD can act as an endogenous antioxidant enzyme and possessed a certain of antioxidant capacity [41, 42]. In normal state, cells would produce more SOD to remove superoxide anions, reflected by the increase of the corresponding gene expression. With the release of zinc ions from Zn-BTC, the activity of intracellular Cu/Zn SOD was increased, leads to the improvement of superoxide scavenging efficiency [25, 26], which was finally manifested as the relatively low expression of SOD expression and inflammatory indexes. The intracellular superoxide anion levels were analyzed using superoxide anion fluorescent probe. As shown in Fig. 4A, different from blank group, the fluorescence intensity of H_2_O_2_ treated group (control group) increased significantly, indicating that the intracellular superoxide anion level increased significantly under by stimulation. Apart from the ineffective influence of H_3_BTC, the introduction of Zn-BTC apparently reduced the fluorescent intensity of H_2_O_2_ treated fibroblasts. The fluorescence intensity was then quantified by Image J (Fig. 4B). The average intensity of blank group was around 12.41 while it became 37.25, 33.70 and 22.01 for control group, H_3_BTC and Zn-BTC, respectively. It was obviously observed that Zn-BTC significantly lowers the intracellular superoxide anion levels.

Besides, the gene expression of H_2_O_2_ induced fibroblasts after treatment was also investigated by qRT-PCT. As shown in Fig. 4C, the gene expression of superoxide dismutase 1 (SOD1) (i), superoxide dismutase 2 (SOD2) (ii) and IL6 (iii) for control group (H_2_O_2_ stimulation) was significantly higher than that of blank group (normal cell). H_2_O_2_ stimulation significantly increased the expression of antioxidant genes to against the damage of oxidative stress. However, H_3_BTC had no effects on the antioxidant gene expression while Zn-BTC presented the apparent decrease of gene expression. The existence of Zn^2+^ was helpful to the decrease of antioxidant gene expression. In the meantime, the gene expression of wound healing was illustrated in Fig. 4D. It was observed that the gene expression of transforming growth factors-β (TGF-β) (i) and COL1 (ii) was relatively low for control group (normal untreated cell) and H_3_BTC. However, Zn-BTC significantly increased both wound healing gene expression after co-culture. As it known to all, COL1, an important extracellular matrix of skin and with its synthesis promoted by TGF-β, is of great significance in skin wound repair [38]. It also confirmed that Zn^2+^ contributed to the wound healing by improving the wound healing genes expression, which was also approved by other research work [43, 44]. Besides, the Cu/Zn SOD activity was also investigated to evaluate the status of intracellular antioxidant system. As shown in Fig. 4E, Zn-BTC could significantly increase the level of intracellular Cu/Zn SOD activity, indicating that the introduction of Zn^2+^ could improve the function of Cu/Zn SOD that inhibited by H_2_O_2_.

### Antibacterial ability of Zn-BTC

**Figure 5.**
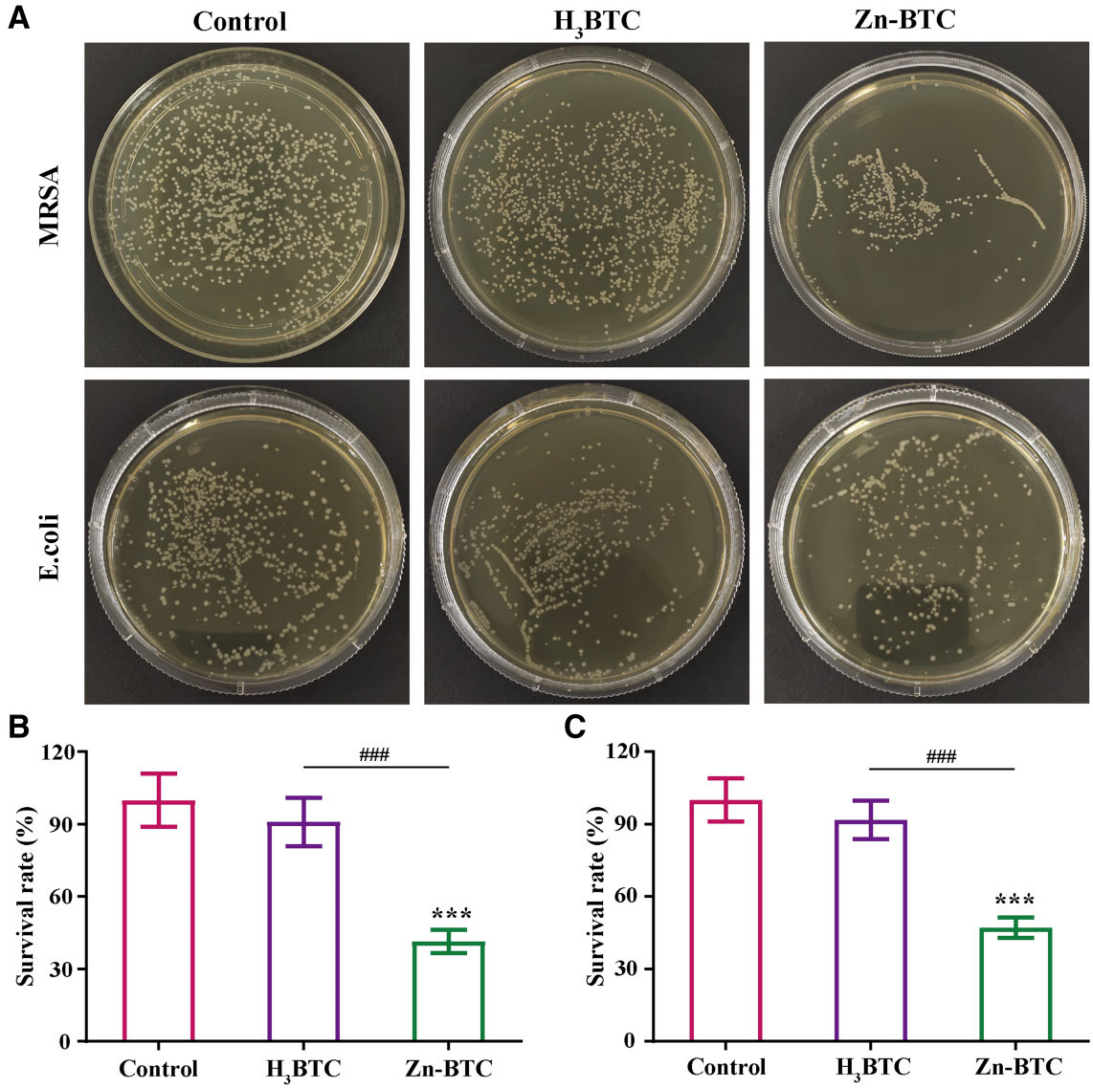
Antibacterial ability of Zn-BTC against MRSA and *E.coli*: bacteriostatic results (**A**) and the corresponding quantified results (**B** and **C**) (‘^*^’ symbol compared with control group, ^***^P < 0.001, and ‘^#^’ symbol compared with H_3_BTC group, ^###^*P* < 0.001)

Co-culture method was often applied to investigate the antibacterial ability of drugs or materials. The quantitative comparison of antibacterial properties can be carried out by calculating the colony on the surface of the culture medium. As shown in Fig. 5A, the number of bacteria decreased significantly on Zn-BTC, indicating that the introduction of Zn^2+^ inhibited the growth of MRSA and *E.coli*. The quantified results of antibacterial ability were listed in Fig. 5B and C. It was found that the antibacterial ability of H_3_BTC was limited compared to control group while the colony count of MRSA and *E.coli* reduced to around 41.4% and 47.2%, respectively, for Zn-BTC, indicating the favorable antibacterial effect of Zn-BTC.

### *In vivo* wound healing effects

**Figure 6.**
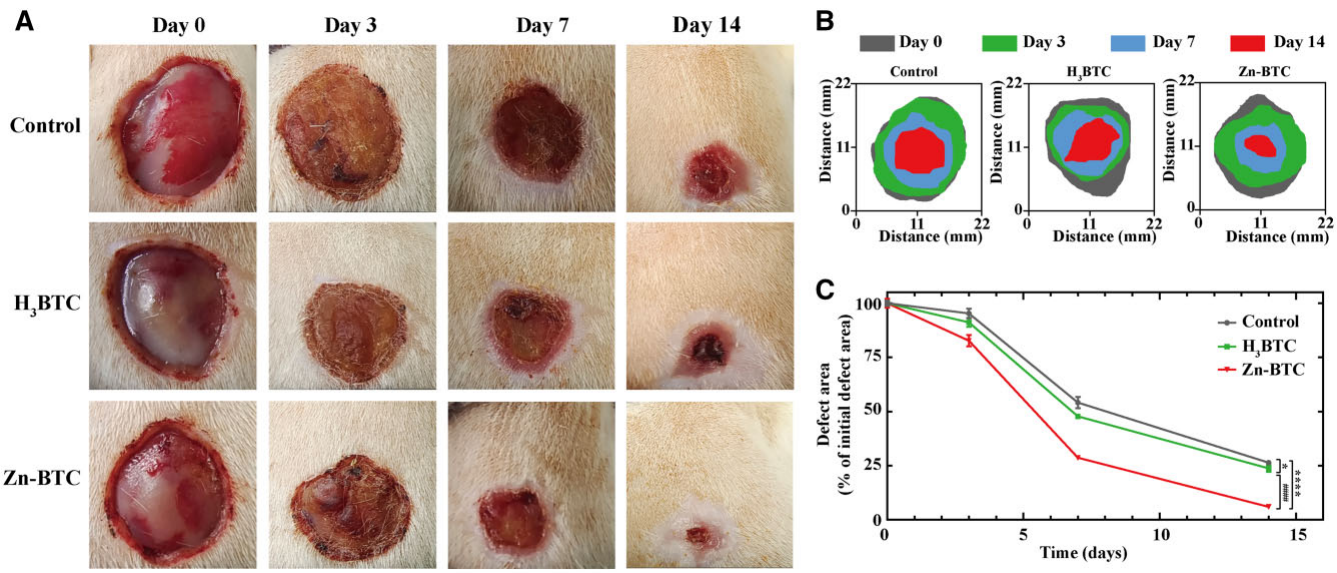
*In vivo* wound healing effects. (**A**) Macroscopic images of wound area treated with different materials at Days 0, 3, 7 and 14, and their corresponding schematic image (**B**) and statistic results (**C**) of wound area changes versus time (‘^*^’ symbol compared with control group, ^*^P < 0.05 and ^****^P < 0.0001, and ‘^#^’ symbol compared with H_3_BTC group, ^####^*P* < 0.0001)

Wound infection model was established using MRSA to infect wound area of SD rats. The wound area was observed at Days 0, 3, 7 and 14, respectively, after therapy. It was obviously observed that Zn-BTC expressed the optimum wound healing effect followed by the order of H_3_BTC and control group (Fig. 6A and C). In Fig. 6B, it schematically displayed the change of wound area versus time, which also clearly reflected the best healing effects of Zn-BTC among them. By comparing the wound size at each time point with the initial wound size, the percentage of remaining wound size was applied to quantify the wound healing effect. Zn-BTC presented the best healing effect at Days 0, 3, 7 and 14. At Day 14, Zn-BTC could improve the wound healing with only around 6.0% wound area left, which was 23.7% and 26.4% for H_3_BTC and control group, respectively (Fig. 6C). From the above, the acceleration of wound healing was directly related to the antibacterial effect of materials.

### *In vivo* histological evaluation

**Figure 7.**
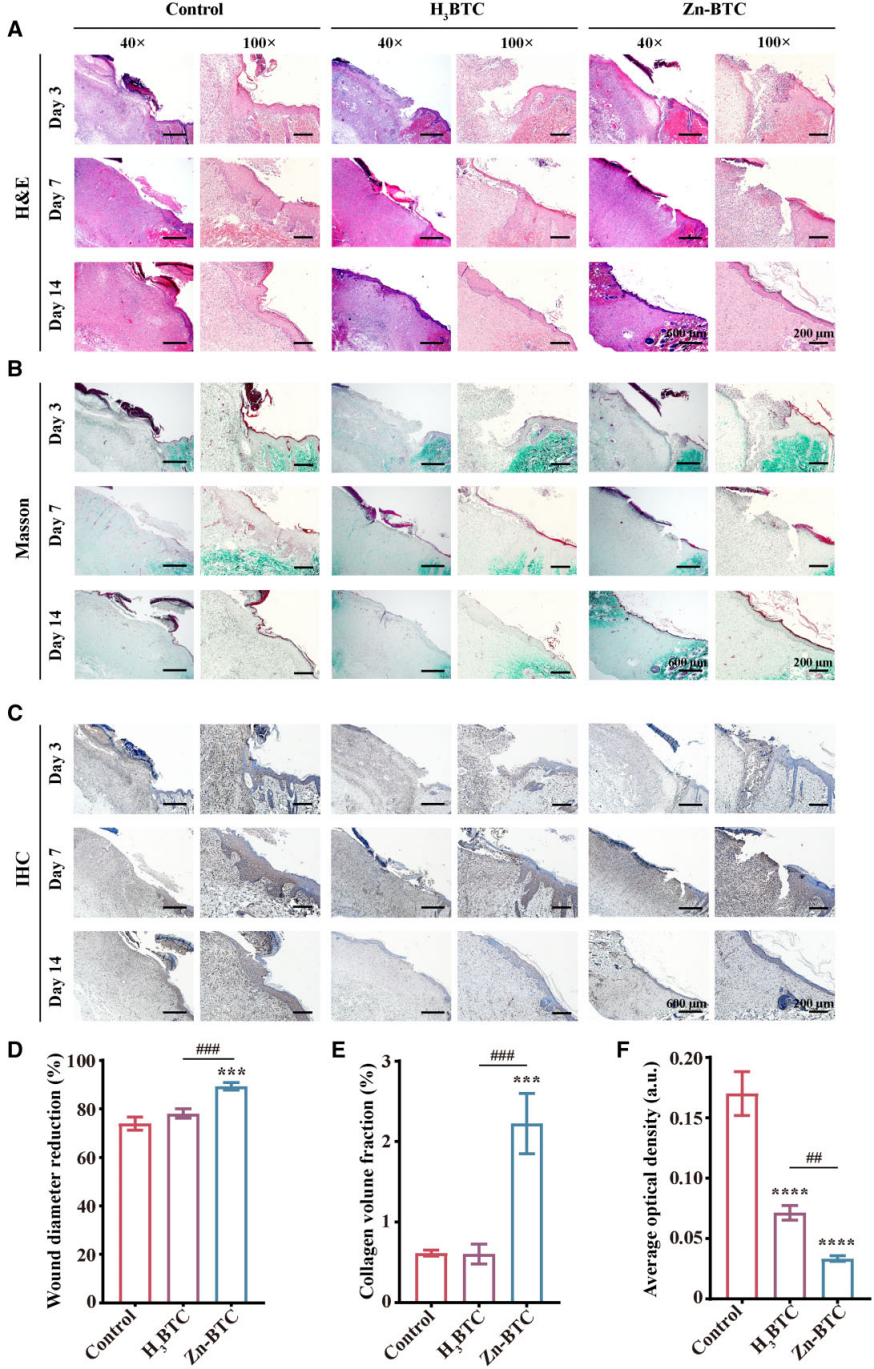
Histological evaluation of skin wound healing. (**A**) H&E staining results of skin wound after treatment at Days 3, 7 and 14, respectively, and the corresponding wound diameter reduction ratio at Day 14 (**D**). (**B**) Masson staining results of skin wound after treatment at Days 3, 7 and 14, respectively, and the corresponding collagen volume fraction at Day 14 (**E**). IHC staining results of skin wound after treatment at Days 3, 7 and 14, respectively (**C**), and the corresponding IHC staining density at Day 14 (**F**) (bar =600 µm for the magnification of 40×, and bar =200 µm for the magnification of 100×) (‘^*^’ symbol compared with control group, ^***^P < 0.001 and ^****^P < 0.0001, and ‘^#^’ symbol compared with H_3_BTC group, ^##^*P* < 0.01 and ^###^*P* < 0.001)

The histological evaluation of wound healing was implemented by H&E, Masson and IHC staining. Figure 7A illustrated the H&E staining results of skin wound at Days 3, 7 and 14. At Day 3, the acute inflammation was observed for all groups with inflammatory cells and disappeared epidermis tissue. However, the tissue of Zn-BTC possessed fewer infiltrated inflammatory cells and more fibroblasts than that of control group and H_3_BTC at Day 7. Furthermore, only basic components of epithelium and dermis formed in control group and H_3_BTC while the obvious repaired epithelial and dermal tissues, and hair follicles occurred for Zn-BTC. The wound diameter from H&E staining slices at Day 14 was also measured to evaluate wound healing (Fig. 7D). Similar to the macroscopic results of skin wound, the wound diameter in Zn-BTC significantly reduced compared to control group and H_3_BTC.

Besides, the Masson staining of skin tissue was illustrated in Fig. 7B. The collagen was observed in green color by Fast Green staining. Thus, the effect of promoting collagen production in each group could be evaluated by quantifying the degree of collagen deposition in the wound defect. At Day 14, compared with the slight green staining of other groups, Zn-BTC presented significantly deep green, indicating the deposition of much collagen. The collagen volume fraction at Day 14 was analyzed by Image J (Fig. 7E). After calculation, the collagen volume fraction was around 2.22% for Zn-BTC, significantly higher than that of control group (0.62%) and H_3_BTC (0.60%).

Finally, the immunohistochemistry staining was applied to confirm the anti-inflammatory effect of Zn-BTC. The IHC staining was illustrated in Fig. 7C. It was found that the intensity of IL6 was weak for H_3_BTC and Zn-BTC than that of control group, especially at Days 3 and 14. After quantified analysis by Image J software, the inflammatory response of wound epithelium in Zn-BTC was smaller than that of H_3_BTC and control group at Day 14 (Fig. 7F).

Therefore, Zn-BTC presented the best healing effects of epithelial and dermal tissues with more visible hair follicles and blood vessels compared to other groups. The above results further confirmed that Zn-BTC could effectively promote the skin wound healing, providing a new strategy for preparing skin repair dressing.

### *In vivo* toxicity evaluation

**Figure 8.**
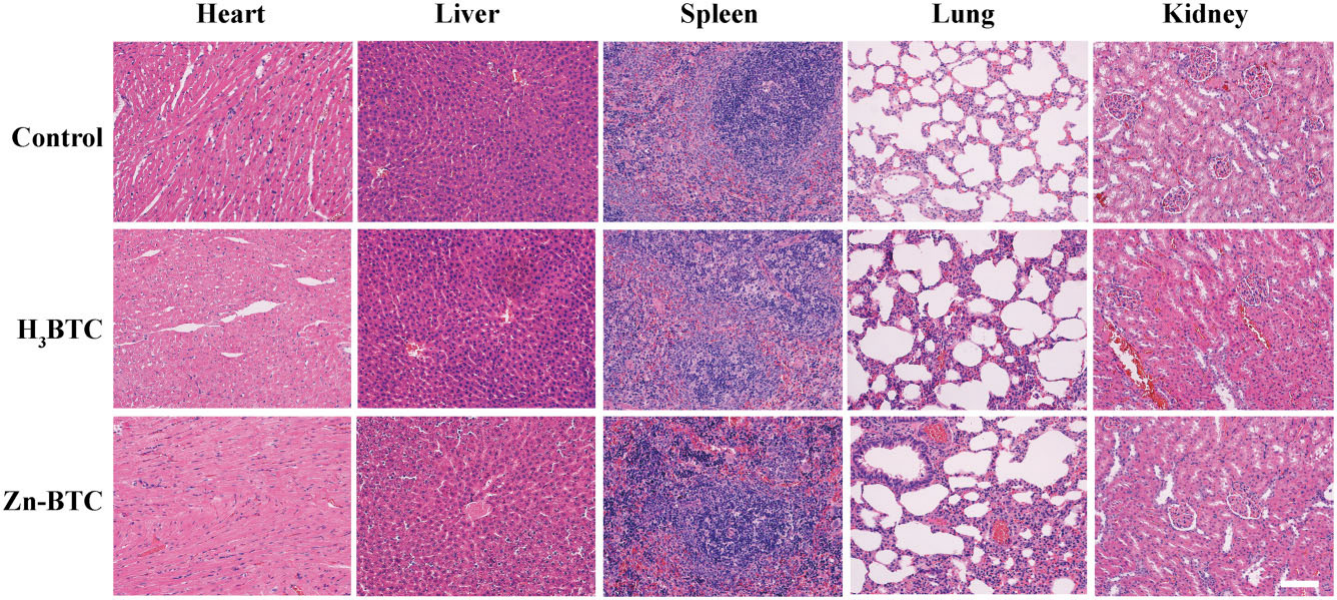
*In vivo* toxicity of Zn-BTC. The H&E staining results of major organs (heart, liver, spleen, lung and kidney) at Day 14 after wound treatment (bar = 200 µm)

The *in vivo* toxicity of Zn-BTC was also evaluated by pathological methods. Figure 8 exhibited the H&E staining of tissue sections of major organs after wound treatment at Day 14. For Zn-BTC, there were no significant histopathologic changes, indicating that no histological damages happened to all organs compared to control group. From the above results, it displayed that Zn-BTC could not bring obvious biological toxicity when applied for skin administration.

## Conclusion

In this study, the Zn based MOF (Zn-BTC) was developed to promote the skin wound healing through its unique antioxidant, anti-inflammatory and antibacterial capacity. Zn-BTC had confirmed its porous and stable framework structures with a rod-like shape in hundreds nm by physicochemical characterization. The rapid degradation of Zn-BTC happened at weak acidic conditions (pH = 5.5) while it achieved slow release of Zn^2+^ in physiological environment. In cellular level, Zn-BTC could promote the migration and proliferation of fibroblasts, and improve the antioxidant and anti-inflammatory capacity as well as wound healing effects, with the low expression of antioxidant genes and high expression of wound healing genes. Besides, it also demonstrated that Zn-BTC had significantly improved antibacterial ability with low cytotoxicity. Finally, Zn-BTC presented the favorable wound healing effects and could not induce the toxicity of major organs *in vivo*. This study paved the way for developing the Zn-based MOF with antibacterial and anti-inflammatory properties further to promote skin wound healing.

## Author contributions

Y.C., J.C. and D.L. participated in the investigation, data processing and analysis, experiment management and article writing. S.L. and D.L. participated in the data processing and analysis. Y.C., J.C., D.L. and M.G. participated in revising the manuscript. L.Z., Q.W. and M.G. as corresponding authors, acquired the funding, participated in the study conception, data analysis and reviewing and finalized this article. All authors have read and agreed to the published version of the article.

## Funding

The work was financially supported by National Natural Science Foundation of China (Grant No. 82160430), the Guangxi Science and Technology Base and Talent Special Project (Grant No. GuikeAD21075002, GuikeAD19254003), the Guangxi Scientific Research and Technological Development Foundation (Grant No. GuikeAB21220062), the Natural Science Foundation of Guangxi (Grant No. 2020GXNSFAA159134) and the Nanning Qingxiu District Science and Technology Major Special Project (Grant No. 2020013).

## Institutional review board statement

Animal care conformed to institutional guidelines. All animal studies were agreed by the Animal Ethics Committee of Guangxi Medical University.

*Conflict of interest statement*. None declared.

## Data availability

The data presented in this study are available on request from the corresponding authors.
